# Low expression of hsa-miR-34c-5p in sperm is associated with unexplained recurrent miscarriage

**DOI:** 10.3389/fgene.2026.1815185

**Published:** 2026-05-29

**Authors:** Hui Tian, Xiao Xi Zhao

**Affiliations:** 1 First Clinical Medical College, Inner Mongolia Medical University, Hohhot, China; 2 Department of Gynecology and Obstetrics, Affiliate Hospital of Inner Mongolia Medical University, Hohhot, China

**Keywords:** bioinformatic analysis, hsa- miR-34c-5p, Notch signalling pathway, spermatozoal miRNAs, unexplained recurrent miscarriages

## Abstract

**Background:**

Recurrent miscarriage affects up to 1%–2% of reproductive-age couples, with nearly 50% of cases classified as unexplained recurrent miscarriage after comprehensive clinical workup. Current etiological research focuses heavily on maternal factors, while the contribution of paternal factors—especially sperm-borne miRNAs, key epigenetic regulators of preimplantation embryonic development—remains understudied. This study aimed to profile differential sperm miRNA expression in partners of women with unexplained recurrent miscarriage, and explore its association with unexplained recurrent miscarriage.

**Methods:**

We enrolled 22 partners of women with unexplained recurrent miscarriage (case group) and 19 healthy males undergoing routine preconception screening (control group) at the Affiliated Hospital of Inner Mongolia Medical University between July 2021 and February 2023. Sperm miRNAs were profiled via high-throughput sequencing, followed by RT-qPCR validation of 3 paternal miRNAs in embryos. GO and KEGG enrichment analyses were performed for target genes of key candidate hsa-miR-34c-5p.

**Results:**

We identified 90 differentially expressed miRNAs in case group sperm, 4 of which were paternal miRNAs in embryos. RT-qPCR confirmed significant downregulation of sperm hsa-miR-34c-5p in cases (p = 0.001). Logistic regression identified low expression of hsa-miR-34c-5p in sperm was significantly associated with unexplained recurrent miscarriage (OR = 4.344, 95%CI 1.119–16.857, p < 0.05), with a ROC-AUC of 0.836. Its target genes were enriched in cellular membrane components, nervous system-related processes, Notch and Rap1 signaling pathways.

**Conclusion:**

This study reveals the association between low sperm miR-34c-5p expression and unexplained recurrent miscarriage, further advancing our understanding of sperm-derived small RNAs in regulating embryonic development and pregnancy outcomes. Nevertheless, the limited sample size means our findings require validation in large-scale multicenter cohorts.

## 1 Introduction

Recurrent miscarriage (RM), defined as two or more consecutive pregnancy losses, affects approximately 1%–2% of reproductive-age couples globally (Van Wely, 2023; Yu et al., 2023). Despite being a prevalent multifactorial reproductive disorder, the etiopathogenesis of RM remains incompletely elucidated, and accurate incidence estimation is hindered by heterogeneous diagnostic criteria across regions (Van Wely, 2023; Yu et al., 2023). Current standard clinical workup for RM includes systematic assessments for uterine anomalies, endocrine disorders, immune dysfunction, thrombophilia, and parental chromosomal abnormalities (Papas and Kutteh, 2020). Even after comprehensive evaluations, however, the underlying cause of pregnancy loss remains unidentified in nearly 50% of affected couples, a condition termed unexplained RM (URM) (Linehan et al., 2023). The absence of a definitive etiology precludes nearly half of RM patients from accessing targeted treatment strategies, imposing severe psychological distress and economic burden on affected individuals and their families (Quenby et al., 2021). Notably, existing clinical and research efforts for RM have been heavily centered on maternal pathogenic factors, while the contribution of paternal factors to URM remains largely understudied.

It is increasingly recognized that the paternal role in embryonic development extends far beyond the provision of 50% of the diploid genomic DNA. During fertilization, spermatozoa deliver a complex repertoire of functional biomolecules into the oocyte, including a diverse population of coding and non-coding RNAs (Joshi et al., 2023). Among these, small non-coding RNAs (sncRNAs) – predominantly microRNAs (miRNAs), Piwi-interacting RNAs (piRNAs), and transfer RNA fragments (tRFs) – have emerged as key epigenetic regulators of early embryonic development (Joshi et al., 2023). miRNAs are a class of evolutionarily conserved, short (20–23 nucleotide) single-stranded non-coding RNAs that mediate post-transcriptional gene silencing via complementary binding to target mRNAs, thereby inhibiting translation or inducing mRNA degradation (Cohen et al., 2024). Through this regulatory mechanism, miRNAs govern a wide spectrum of fundamental biological processes, including gametogenesis, preimplantation embryonic development, and cell fate determination (Olotu et al., 2023).

Sperm sncRNAs are dynamically generated during spermatogenesis and post-testicular maturation in the epididymis, and their expression profiles are highly susceptible to modulation by environmental and physiological factors (Vallet-Buisan et al., 2023; Salilew-Wondim et al., 2020). Upon fertilization, sperm-borne sncRNAs are transferred into the oocyte, where they persist into the preimplantation embryonic stage and regulate zygotic genome activation and early embryonic gene expression, ultimately shaping offspring developmental and phenotypic outcomes (Vallet-Buisan et al., 2023; Salilew-Wondim et al., 2020).

Functional evidence from murine models has firmly established the essential role of sperm miRNAs in normal embryonic development. In 2016, Yuan et al. (2016) demonstrated that sperm from mice with conditional knockout of *Dicer* and *Drosha*–the two core enzymes responsible for miRNA biogenesis and maturation–failed to support normal embryonic development after ICSI. Strikingly, microinjection of total RNA from wild-type sperm into the developmentally arrested embryos fully rescued their developmental potential. In a subsequent 2017 study, Guo et al. (2017) further validated this finding: depletion of ∼90% of endogenous RNA from mature mouse spermatozoa resulted in drastically reduced blastocyst formation and embryo survival rates after ICSI, while supplementation with wild-type sperm RNA completely reversed these developmental defects. These landmark studies provide causal evidence that sperm-borne RNAs, particularly miRNAs, are indispensable for early embryonic development and survival.

Beyond embryonic developmental competence, sperm miRNAs have also been implicated in intergenerational epigenetic inheritance of phenotypic traits. Two independent studies by Fullston et al. (2013) and Grandjean et al. (2015) showed that male mice fed a high-fat diet developed obesity and metabolic dysfunction, with concurrent significant alterations in the sperm miRNA signature. These dysregulated sperm miRNAs were transmitted to the oocyte during fertilization, driving aberrant embryonic gene expression and metabolic programming, which ultimately led to the inheritance of obesity and metabolic disorders in the offspring. These findings highlight that sperm miRNAs can act as epigenetic carriers of paternal environmental exposures, with profound impacts on embryonic development and long-term offspring health.

In humans, Krawetz et al. (2011) first characterized the sncRNA landscape of human spermatozoa via high-throughput sequencing, identifying an abundance of miRNAs with predicted regulatory roles in early embryonic development. Comparative analysis of miRNA profiles across human sperm, oocytes, and preimplantation embryos further identified four miRNAs (hsa-miR-34c, hsa-miR-375, hsa-miR-25, and hsa-miR-252) that were exclusively present in sperm and embryos but undetectable in oocytes, confirming their exclusive paternal origin in the early embryo (Krawetz et al., 2011). The same study also identified a panel of sperm-borne paternal miRNAs involved in epigenetic regulation via DNA methylation, including hsa-miR-140, hsa-miR-21, hsa-miR-152, and hsa-miR-148a (Krawetz et al., 2011). Paternal miRNAs in embryos, specifically referring to the miRNAs delivered by sperm and present in the early embryo. Collectively, existing evidence demonstrates that sperm miRNAs are essential regulators of early embryonic development, and that dysregulation of sperm miRNA expression is closely linked to adverse developmental and reproductive outcomes. However, the association between sperm miRNA dysregulation and URM remains incompletely characterized.

Against this backdrop, the present study aimed to systematically characterize the differential expression profile of sperm miRNAs in partners of women with URM, and to explore the potential association between aberrant sperm miRNA expression and URM occurrence.

## 2 Methods

### 2.1 Ethical approval and informed consent

This study was conducted in strict accordance with the ethical principles of the Declaration of Helsinki, and was approved by the Medical Ethics Committee of Inner Mongolia Medical University (Hohhot, China; Ethics Approval No.: YKD202301105). Informed consent was obtained from all participants prior to study enrollment.

### 2.2 Study design and settings

This study employed a case-control design. Semen samples were collected from the partners of women with URM treated at the Affiliated Hospital of Inner Mongolia Medical University, and from healthy men undergoing physical examinations between July 2021 and February 2023.

### 2.3 Participants

#### 2.3.1 Case group

##### 2.3.1.1 Inclusion criteria

Male participants were eligible for the case group if their female partner had a history of ≥2 consecutive spontaneous pregnancy losses, and no clinically significant abnormalities were identified in either partner after systematic evaluations: the female partner underwent assessments of uterine anatomy, immune function, endocrine status, and coagulation function, while both partners completed chromosomal karyotyping.

##### 2.3.1.2 Exclusion criteria

Participants with systemic acute or chronic diseases, or a prior history of radiotherapy or chemotherapy were excluded from this group.

#### 2.3.2 Control group

##### 2.3.2.1 Inclusion criteria

Male participants were enrolled in the control group if they were in good general health, had at least one healthy live birth, and all conventional semen analysis parameters were within the normal reference range.

##### 2.3.2.2 Exclusion criteria

Participants with systemic acute or chronic diseases, a history of infertility, or a female partner with a history of adverse pregnancy outcomes were excluded.

### 2.4 Data and specimen collection

General clinical and demographic data of all enrolled participants were extracted from the electronic medical case registration system at the time of their outpatient visit. Prior to semen collection, all participants were instructed to maintain sexual abstinence (no ejaculation) for 3–7 days, in accordance with the World Health Organization (WHO) laboratory manual for the examination and processing of human semen. Semen specimens were collected into sterile, labeled containers via masturbation, and immediately transferred to the laboratory for processing. Samples were left to liquefy completely in a 36.6 °C humidified constant-temperature incubator for 30 min. Following complete liquefaction, a 2 mL aliquot was taken from each semen sample for purification via density gradient centrifugation, followed by treatment with somatic cell lysis buffer to eliminate contaminating somatic cells. The purified sperm pellets were immediately snap-frozen in liquid nitrogen and stored at −80 °C until RNA extraction. Total RNA, including the small RNA fraction, was isolated from the purified sperm samples using the miRNeasy Micro Kit (QIAGEN, Hilden, Germany), strictly following the manufacturer’s standard protocol. A 1 µL aliquot of the final eluted RNA was used to quantify RNA concentration and evaluate RNA purity (via the A260/A280 absorbance ratio) using an ultraviolet-visible spectrophotometer.

### 2.5 miRNA sequencing and differential miRNA screening

Total RNA samples obtained as described above were sent to Shanghai OE Biotech. Co., Ltd. for differential miRNA expression profiling via high-throughput next-generation sequencing (NGS). The integrity of total RNA samples was assessed by agarose gel electrophoresis, and RNA quantification and purity evaluation were performed using a NanoDrop ND-1000 ultra-micro spectrophotometer (NanoDrop Corporation, Wilmington, DE, USA). A QIAseq miRNA Library Kit (QIAGEN, Hilden, Germany) was used to construct miRNA sequencing libraries. The qualified libraries were then sequenced on the Illumina NovaSeq 6000 platform (Illumina, San Diego, CA, USA) with a 150 bp paired-end sequencing strategy (2 × 150 bp read length). Raw sequencing data were filtered to obtain high-quality clean reads for subsequent analysis. Comprehensive quality assessment was performed on the clean sequences, including quality metrics, sequence length distribution, and shared common sequences across all samples. To ensure comparability between the case and control groups, miRNA expression levels were normalized using transcripts per million (TPM), calculated with the following formula: TPM = (miRNA read counts/total mapped read counts of the sample) × 10^6^. Differential expression analysis of miRNAs was conducted using the limma package in R software (R Foundation for Statistical Computing, Vienna, Austria). The screening criteria for differentially expressed miRNAs were set as |log_2_ (fold change)| ≥1.5 and p-value <0.05.

### 2.6 RT–qPCR validation of paternal miRNAs in embryos expression levels

Total RNA that passed quality control was subjected to polyadenylation and complementary DNA (cDNA) synthesis strictly following the manufacturer’s protocol of the Mir-X miRNA First-Strand Synthesis Kit (Takara Bio, Shiga, Japan). The quantitative real-time PCR (RT-qPCR) reaction system was prepared according to the manufacturer’s instructions of the ChamQ Universal SYBR qPCR Master Mix (Vazyme Biotech, Nanjing, China). Human U6 small nuclear RNA (U6 snRNA) was used as the endogenous reference for normalization, and the relative expression levels of target miRNAs were calculated using the 2^-ΔΔCt^ method. The RT-qPCR amplification conditions were set as follows: initial pre-denaturation at 95 °C for 30s, followed by 40 amplification cycles of denaturation at 95 °C for 10s and annealing/extension at 60 °C for 30s. After amplification, melting curve analysis was performed to verify the specificity of PCR products, with the conditions: 95 °C for 15s, 60 °C for 60 s, and a final gradient heating to 95 °C for 15s with continuous fluorescence acquisition. The primer sequences of target miRNAs and the endogenous reference are listed below (all sequences are presented in the standard 5′ to 3′ direction):

hsa-miR-375-3p: AGTTTGTTCGTTCGGCTC
hsa-miR-34c-5p: AGG​CAG​TGT​AGT​TAG​CTG​ATT​GC
hsa-miR-25-3p: CAT​TGC​ACT​TGT​CTC​GGT​CTG​A
Human U6 snRNA: GGA​ACG​ATA​CAG​AGA​AGA​TTA​GC

### 2.7 Gene Ontology (GO) functional enrichment and Kyoto Encyclopedia of Genes and Genomes (KEGG) pathway enrichment

Analysis Experimentally validated target genes of hsa-miR-34c-5p were retrieved from the human miRTarBase 8.0 database. GO functional enrichment analysis and KEGG signaling pathway enrichment analysis were subsequently conducted on the identified target genes using the ClusterProfiler package (version 4.6.0, Bioconductor) in R software (version 4.3.2; R Foundation for Statistical Computing, Vienna, Austria). GO enrichment analysis was performed across three core ontologies: biological process (BP), cellular component (CC), and molecular function (MF). The Benjamini–Hochberg method was applied for multiple testing correction, and terms with an adjusted p-value (p.adjust) < 0.05 were defined as statistically significantly enriched.

### 2.8 Statistical analysis

Statistical analyses were performed using SPSS version 29.0 software (SPSS Inc., Chicago, IL, USA). The Shapiro-Wilk test was applied to assess the normality of all continuous data distributions. Normally distributed data, including age, sperm concentration, and total sperm motility, are presented as mean ± standard deviation (SD), and intergroup differences were compared using the independent samples Student’s t-test. Non-normally distributed data, including total RNA concentration and the relative expression levels of hsa-miR-375, hsa-miR-34c-5p, and hsa-miR-25, are presented as median with interquartile range (IQR), and intergroup comparisons were performed using the Mann-Whitney U test. Binary logistic regression analysis was conducted to explore the independent association between sperm hsa-miR-34c-5p expression and the occurrence of URM. Receiver operating characteristic (ROC) curve analysis was performed to assess the predictive performance of sperm hsa-miR-34c-5p expression for identifying URM. Post hoc statistical power analysis was performed using G*Power version 3.1 software to verify the reliability of our statistical results. Statistical significance was set at a two-sided p-value <0.05.

## 3 Results

### 3.1 Participants

The case group consisted of 22 partners of women with a history of ≥2 consecutive spontaneous miscarriages, who had undergone comprehensive systematic clinical evaluations with no identifiable underlying etiology for pregnancy loss. The control group included 19 healthy men who presented for routine preconception physical examinations, with all conventional semen analysis parameters within the normal reference range. The age range of participants in both the case and control groups was 25–45 years.

### 3.2 Differentially expressed miRNAs in sperm from the partners of women with URM

The comparisons of baseline demographic and clinical characteristics and sperm quality parameters between the case and control groups are summarized in Table 1. A total of 90 significantly differentially expressed miRNAs (DEMs) were identified between the case and control groups, among which 42 were significantly upregulated and 48 were significantly downregulated (Table 2). Four of these 90 DEMs have been previously documented in the literature as paternal miRNAs in embryos (Table 2).

**TABLE 1:** Metadata and sperm characteristics of the case and control group.

Index	Sequencing sample		Total sample		*t*	*p*-value
	Case	Control	Case	Control		
	*n* = 3	*n* = 3	*n* = 22	*n* = 19		
Age (years)	34.66 ± 2.31	35.00 ± 2.00	34.63 ± 4.88	33.10 ± 3.73	1.114	0.272
Sperm concentration (%)	37.71 ± 5.27	38.63 ± 12.66	51.88 ± 27.34	52.64 ± 20.38	−0.1	0.921
Total sperm motility (%)	56.69 ± 16.70	62.54 ± 9.08	50.09 ± 15.90	66.39 ± 9.78	−1.797	0.081
DNA integrity (%)	88.33 ± 1.53	89.44 ± 3.00	​	​	​	​

**TABLE 2:** DEMs in sperm from the partners of women with URM.

Index	miRNA ID
Upregulated	hsa-let-7a-5p, -7c-5p, -7d-5pI, -7e-5p, hsa-miR-103b, −107, −125b-5p, −1269a, -134-5p, −135a-2-3p, -141-3p, -141-5p, −193a-3p, −193b-5p, −200a-3p, −200b-3p, −200c-3p, −203a-3p, −203b-5p, -210-3p, −23b-3p, −26b-5p, −29a-3p, −29b-3p, −29c-3p, -3184-3p, −320b, -331-5p, -361-5p, −374b-5p, -**375-3p**, −3911, -412-5p, −429, -4664-3p, -574-3p, -676-3p, -744-5p, hsa-miR-7705, -99a-5p, **-148a-5p**, -363-3p
Downregulated	hsa-miR-526a-5p, hsa-miR-520c-5p, hsa-miR-518d-5p, hsa-miR-1-3p, -1224-5p, -1285-3p, -132-3p, -139-5p, **-140-5p**, -143-3p, -145-3p, −146b-3p, −146b-5p, −1843, −196a-5p, −199b-5p, -212-5p, −219a-2-3p, −3148, -335-3p, −3690, -3912-3p, -3925-5p, -4485-3p, −451a, -4661-5p, −4686, −4705, -486-3p, -486-5p, −499a-5p, −518a-3p, −520g-5p, -525-5p, −548am-3p, −548k, −548l, −548o-3p, −548u, -552-3p, −5683, -625-3p, -6500-3p, -6874-3p, -9-5p, −30a-3p, **-34c-5p**, -3529-3p

Paternal miRNAs, in embryos are marked in bold text.

### 3.3 Larger-sample qPCR validation of paternal miRNAs in embryos (hsa-miR-375-3p, hsa-miR-34c-5p, hsa-miR-25-3p) expression

Conventional semen parameters and the relative expression levels of the three selected target miRNAs (hsa-miR-34c-5p, hsa-miR-375-3p, and hsa-miR-25-3p) in the case and control groups are summarized in Table 3. Consistent with our high-throughput sequencing results, the relative expression level of hsa-miR-34c-5p was significantly downregulated in the case group compared with the control group (*p* = 0.001). However, no statistically significant intergroup differences were detected in the relative expression levels of hsa-miR-375-3p (*p* = 0.985) or hsa-miR-25-3p (*p* = 0.545).

**TABLE 3:** Semen parameters and expression levels of miRNAs in case and control groups.

Index	Case (*n* = 19)	Control (*n* = 16)	t/Z	*p*-value
Age (years)	34.63 ± 5.22	32.75 ± 3.91	−1.188	0.243
Sperm concentration (10^6^/mL)	54.11 ± 28.80	55.27 ± 20.75	0.134	0.894
Total sperm motility (%)	59.46 ± 16.21	67.12 ± 10.01	1.641	0.110
RNA concentration (ng/µL)	145.71 ± 63.78	193.70 ± 89.18	−1.424	0.154
hsa-miR-34c-5p	0.56 ± 0.61	1.39 ± 1.01	−3.378	0.001^※^
hsa-miR-375-3p	1.81 ± 2.29	1.23 ± 1.71	−0.038	0.970
hsa-miR-25-3p	2.76 ± 2.70	2.05 ± 1.78	−0.632	0.527

※Statistically significant difference.

### 3.4 To assess the association between sperm hsa-miR-34c-5p expression and URM

Binary logistic regression analysis was conducted to explore the association between sperm hsa-miR-34c-5p expression and the occurrence of URM. The results confirmed that low expression of hsa-miR-34c-5p in sperm was significantly associated with unexplained recurrent miscarriage (Table 4). AUC of the ROC curve was 0.836.

**TABLE 4:** Logistic regression analysis results of miR-34 expression level in sperm and risk of URM.

​	​	​	​	​	​	OR (95% CI)	
Index	B	SE	Wals	*p*-value	OR	Lower	Upper
Age	−0.078	0.122	0.412	0.521	0.925	0.727	1.175
Sperm concentration	0.002	0.019	0.007	0.936	1.002	0.965	1.04
Total sperm motility	−0.05	0.037	1.832	0.176	0.951	0.884	1.023
miRNA concentration	−0.007	0.007	1.238	0.266	0.993	0.98	1.006
hsa-miR-34c-5p	−1.799	0.876	4.218	0.04※	0.166	0.03	0.921
Constant	8.771	6.53	1.804	0.179	6442.484	​	​

※Statistically significant difference.

B, partial regression coefficient; CI, confidence interval; OR, odds ratio; SE, standard error of the partial regression coefficients.

### 3.5 Bioinformatics analysis of hsa-mir-34c-5p

**FIGURE 1:**
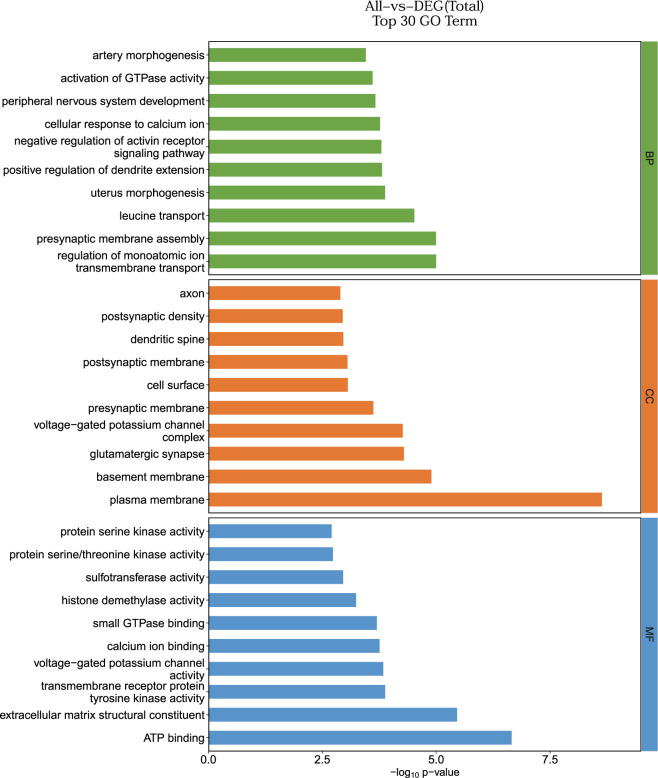
Gene Ontology (GO) enrichment map of microRNA (miR)-34c-5p. The miR-34c-5p gene set is significantly enriched in biological processes, including artery morphogenesis and the activation of GTPase activity. Regarding cellular composition, the genes are primarily associated with functional cellular structures, including cell membranes and nervous system components, such as axons, postsynaptic densities, and presynaptic membranes. Molecular function enrichment analysis revealed that these genes participate in molecular activities such as ATP binding and protein serine/threonine kinase activity. DEG, differentially expressed gene.

**FIGURE 2:**
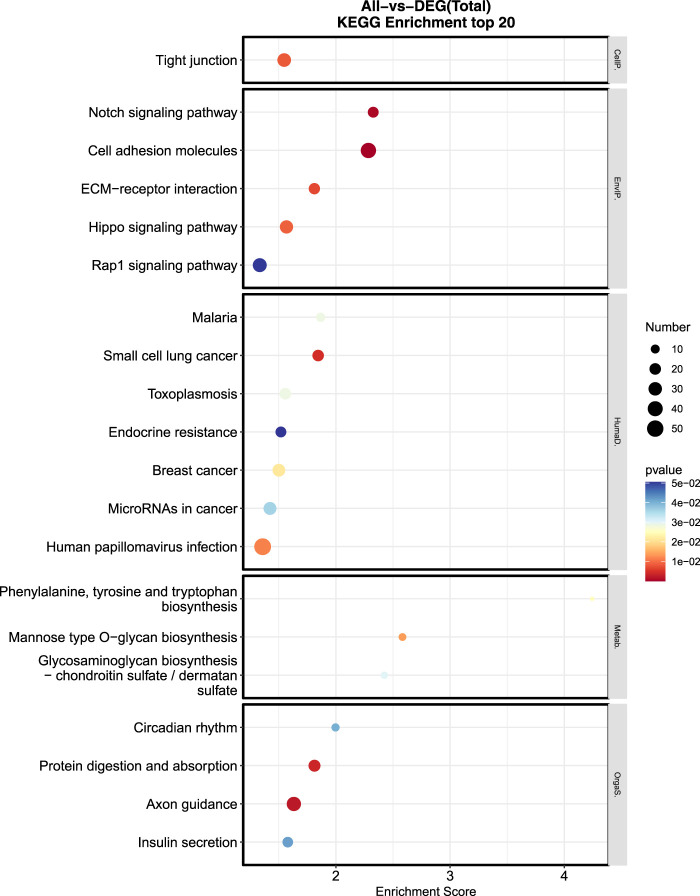
Kyoto Encyclopedia of Genes and Genomes (KEGG) pathway enrichment analysis of miR-34c-5p target genes. The top 20 enriched KEGG pathways associated with the target genes of miR-34c-5p are shown, including the cell tight junction, Notch signalling pathway, Rap1 signalling pathway, and cell adhesion molecules, as well as metabolic pathways such as phenylalanine biosynthesis. Source: Kanehisa Laboratories, KEGG: Kyoto Encyclopedia of Genes and Genomes, https://www.kegg.jp/ (Kanehisa et al., 2016; Kanehisa and Goto, 2020).

Figures 1, 2 present the results of GO functional enrichment annotation and KEGG pathway enrichment analysis for the target genes of hsa-miR-34c-5p, respectively. The target genes were significantly enriched in core biological processes related to developmental programs, including nervous system development and cellular signaling pathways. For cellular component annotation, the most enriched terms were associated with neuronal structural components (axons and synapses). The enriched molecular function terms mainly included ATP binding, signal transduction activity, and protein kinase activity. KEGG pathway analysis revealed that the target genes of hsa-miR-34c-5p were mainly enriched in key pathways, including the tight junction, Notch signaling pathway, Rap1 signaling pathway, cell adhesion molecules, and phenylalanine biosynthesis pathways.

### 3.6 Post hoc power analysis

Between-group comparisons were performed using the two-tailed Mann-Whitney U test for the study group (n = 19) and the control group (n = 16). This analysis revealed a statistically significant difference between groups (U = 50.00, Z = −3.378, exact two-tailed P = 0.01).

Post hoc statistical power analysis was conducted using G*Power 3.1 software to validate the robustness of our findings. Based on the observed effect size (Cohen’s d = 0.99) derived from the current experimental data, the achieved *post hoc* power was 79%, which approximates the well-established 80% threshold for adequate statistical power in biomedical research, supporting the reliability of our between-group comparison results.

In addition, we performed an *a priori* sample size calculation for a subsequent confirmatory trial using our pilot data. The calculation demonstrated that 32 subjects per group would be required to achieve 80% statistical power at a two-sided α level of 0.05. To account for a prespecified 10% dropout rate, the total required sample size was 64 subjects (32 per group).

## 4 Discussion

In this study, high-throughput sequencing combined with RT-qPCR was used to analyze the miRNA expression profiles in sperm from partners of women with URM. A total of 90 DEMs were identified, among which 4 paternal miRNAs in embryos are reported in previous studies. RT-PCR validation confirmed that hsa-miR-34c-5p was downregulated, which was consistent with the high-throughput sequencing results. To investigate the functional role of hsa-miR-34c-5p, we predicted the target genes of hsa-miR-34c-5p in this study, and performed functional annotation, pathway analysis on these genes to elucidate its biological significance.

In this study, no statistically significant differences in routine semen parameters were observed between the case and control groups. However, aberrant miRNA expression profiles were detected in spermatozoa from partners of women with URSA compared with controls. These findings further confirm the limited predictive value of conventional semen parameters for clinical pregnancy outcomes (Sahoo et al., 2021; Krausz et al., 2015), and demonstrate that dysregulated sperm miRNA expression is significantly associated with adverse pregnancy outcomes (Al Smadi et al., 2025), consistent with prior literature in the field.

In this study, three previously reported paternal miRNAs in embryos were selected for RT-PCR validation. Mechanistically, dysregulation of paternal miRNAs in embryos is a core prerequisite for establishing a causal relationship between differentially expressed sperm miRNAs and impaired embryonic development and differentiation. RT-PCR confirmed significant downregulation of hsa-miR-34c-5p in sperm samples of partners of women with URSA, fully consistent with our transcriptomic sequencing results. Accumulating evidence has characterized the key regulatory role of miR-34c in embryonic development (Liu et al., 2012; Cui et al., 2023; Shi et al., 2020), aligning closely with our findings. Taken together, reduced sperm hsa-miR-34c-5p expression predisposes to impaired embryonic development, defective differentiation, and adverse clinical pregnancy outcomes.

Thapliyal et al. (2024) applied the same methodology as our study to profile sperm miRNA expression in male partners of Indian women with URSA, identifying 12 DEMs. Functional enrichment analysis showed that target genes of the upregulated DEMs were mainly enriched in biological processes related to sperm quality control and embryonic development. However, the study did not investigate whether these DEMs are paternally derived miRNAs in the embryo, which is the key difference from our work. Here, we focused specifically on identifying paternal miRNAs in embryos in the DEM profiles, as only verifying the differential expression of these paternal miRNAs in embryos can clarify the direct association between dysregulated sperm miRNA expression and the occurrence of URSA.

To further clarify the functional role of hsa-miR-34c-5p in embryonic development, we conducted GO and KEGG enrichment analyses of its target genes. Our bioinformatic analysis highlighted the Notch signaling pathway as a key regulatory pathway targeted by hsa-miR-34c-5p. This pathway mediates direct cell-cell communication across diverse developmental processes in multiple species, governing cell fate determination, proliferation, and apoptosis (Rokavec et al., 2014; Artavanis-Tsakonas et al., 1999). In vertebrates, Notch signaling is essential for asymmetric cell differentiation, and its gene mutations cause abnormal embryonic development (Schuster-Gossler et al., 2009; Zhang et al., 2002), while developmental defects are a leading cause of spontaneous miscarriage (Shiota, 2021). Murine studies have confirmed that dysregulated Notch signaling impairs somitogenesis and induces neural tube defects (Yoon and Gaiano, 2005), and analyses of aborted human embryos show that 3.6% of early pregnancy losses stem from central nervous system malformations (Creasy and Alberman, 1976). Although no prior studies have directly linked the Notch signaling pathway to recurrent miscarriage, these findings support that hsa-miR-34c-5p may drive early pregnancy loss by regulating Notch signaling. We also identified the Rap1 signaling pathway as another key target of hsa-miR-34c-5p. Rap1 is a small GTP-binding protein activated by extracellular stimuli such as growth factors and cytokines, which is expressed in human placenta, and its knockdown markedly inhibits trophoblast migration and invasion (Huang et al., 2025). Collectively, these pathways and biological processes directly or indirectly modulate post-fertilization events and embryonic development, thereby affecting pregnancy outcomes.

However, this study has several limitations. First, the differential miRNA expression profile was generated via high-throughput sequencing using a discovery cohort of 3 sperm specimens from the URSA group and 3 from the control group. Although we identified 90 DEMs, the subsequent validation results were not fully concordant with the sequencing profile, which is most likely attributable to the limited sample size of the discovery cohort. Second, environmental factors have been reported to modulate sperm miRNA expression levels (Salilew-Wondim et al., 2020). In our study, we did not systematically collect or adjust for data on potential confounding variables, including smoking status, alcohol consumption, and occupation. These methodological constraints may limit the interpretability and generalizability of our findings. Finally, our study did not include *in vitro* functional validation of the identified molecular mechanism.

To further validate our findings, future work should confirm the regulatory effects of downregulated hsa-miR-34c-5p on its target genes and proteins in embryonic cell lines, and delineate the molecular pathway linking reduced miR-34c-5p expression to impaired embryonic cell proliferation and developmental potential. For functional validation of miR-34c-5p, gain- and loss-of-function studies were performed via transfection of miR-34c-5p mimics and inhibitor, respectively. Cell proliferation, apoptosis, migration, and invasion were evaluated using CCK-8, flow cytometry, and Transwell assays. Target gene validation was conducted using dual-luciferase reporter and gene expression correlation analyses.

In addition, to validate the association between low sperm hsa-miR-34c-5p expression and URSA in a larger clinical cohort. Furthermore, the development of a sperm-specific miRNA detection kit could provide a valuable non-invasive tool for the etiological diagnosis of patients with URM.

In conclusion, our study identifies an association between reduced miR-34c-5p expression in sperm and URM, which deepens the understanding of the impacts of sperm-derived small RNAs on embryonic development and reproductive outcomes. However, due to the limited sample size of this cohort, our results need to be further verified in large-scale multicenter studies.

## Data Availability

The datasets supporting the findings of this study are publicly available at: https://doi.org/10.5281/zenodo.20355329.
